# Advanced Spectroscopy and APBS Modeling for Determination of the Role of His190 and Trp103 in Mouse Thymidylate Synthase Interaction with Selected dUMP Analogues

**DOI:** 10.3390/ijms22052661

**Published:** 2021-03-06

**Authors:** Małgorzata Prokopowicz, Adam Jarmuła, Yannick Casamayou-Boucau, Fiona Gordon, Alan Ryder, Justyna Sobich, Piotr Maj, Joanna Cieśla, Zbigniew Zieliński, Piotr Fita, Wojciech Rode

**Affiliations:** 1 Inter-Faculty Interdisciplinary Doctoral Studies in Natural Sciences and Mathematics, MISMaP College, University of Warsaw, ul. Banacha 2C, 02-097 Warsaw, Poland; 2 Institute of Experimental Physics, Faculty of Physics, University of Warsaw, ul. Pasteura 5, 02-093 Warsaw, Poland; Piotr.Fita@fuw.edu.pl; 3 Nencki Institute of Experimental Biology, ul. Pasteura 3, 02-093 Warsaw, Poland; a.jarmula@nencki.edu.pl (A.J.); j.sobich@nencki.edu.pl (J.S.); p.maj@nencki.edu.pl (P.M.); z.zielinski@nencki.gov.pl (Z.Z.); 4 Nanoscale BioPhotonics Laboratory, School of Chemistry, National University of Ireland, University Road, H91 TK33 Galway, Ireland; yannick.casamayou@nuigalway.ie (Y.C.-B.); F.GORDON2@nuigalway.ie (F.G.); alan.ryder@nuigalway.ie (A.R.); 5 Department of Pharmacology, University of Oxford, Mansfield Road, Oxford OX1 3QT, UK; 6 Faculty of Chemistry, Warsaw University of Technology, ul Noakowskiego 3, 00-664 Warsaw, Poland; jciesla@ch.pw.edu.pl

**Keywords:** mouse thymidylate synthase, spectroscopy, APBS modeling

## Abstract

A homo-dimeric enzyme, thymidylate synthase (TS), has been a long-standing molecular target in chemotherapy. To further elucidate properties and interactions with ligands of wild-type mouse thymidylate synthase (mTS) and its two single mutants, H190A and W103G, spectroscopic and theoretical investigations have been employed. In these mutants, histidine at position 190 and tryptophan at position 103 are substituted with alanine and glycine, respectively. Several emission-based spectroscopy methods used in the paper demonstrate an especially important role for Trp 103 in TS ligands binding. In addition, the Advanced Poisson–Boltzmann Solver (APBS) results show considerable differences in the distribution of electrostatic potential around Trp 103, as compared to distributions observed for all remaining Trp residues in the mTS family of structures. Together, spectroscopic and APBS results reveal a possible interplay between Trp 103 and His190, which contributes to a reduction in enzymatic activity in the case of H190A mutation. Comparison of electrostatic potential for mTS complexes, and their mutants, with the substrate, dUMP, and inhibitors, FdUMP and N${}^{4}$-OH-dCMP, suggests its weaker influence on the enzyme–ligand interactions in N${}^{4}$OH-dCMP-mTS compared to dUMP-mTS and FdUMP-mTS complexes. This difference may be crucial for the explanation of the ”abortive reaction” inhibitory mechanism of N${}^{4}$OH-dCMP towards TS. In addition, based on structural analyses and the H190A mutant capacity to form a denaturation-resistant complex with N${}^{4}$-OH-dCMP in the mTHF-dependent reaction, His190 is apparently responsible for a strong preference of the enzyme active center for the *anti* rotamer of the imino inhibitor form.

## 1. Introduction

Thymidylate synthase (TS) is an enzyme with a long-standing history of a dedicated comprehensive research. Its activity was first documented in the 1950s [1]. However, it took over a decade to identify the actual protein molecule and describe, with some uncertainties, its catalytic mechanism [2,3,4,5]. Today we know that this enzyme (EC 2.1.1.45) is quite ubiquitous; it belongs to the most conservative enzymes and catalyses reductive methylation of deoxyuridine monophospate (dUMP) with (6R)-N${}^{5,10}$ methylenetetrahydrofolate (mTHF) as a cofactor, to yield deoxythymidine monophosphate (dTMP) and dihydrofolate (DHF) [6,7].

Nevertheless, after seventy years of investigations, new and surprising facts about the TS catalytic mechanism are still being discovered. Recent findings include for instance:

- Revealing that both reactions leading to dTMP production, (1) methylene group transfer from a cofactor to the C(5) atom of dUMP with the concomitant proton abstraction from C(5) and (2) the hydride transfer from a cofactor to the exocyclic methylene group (donated in reaction (1)), display a higher complexity that lies in a strong liability of the C(6)-γS bond between the thiol of catalytic cysteine and C(6) of dUMP [8,9,10,11,12,13];
- The observation of the enhanced emission quenching of the human TS in comparison to *E. coli* TS by 5-fluoro-dUMP (FdUMP) [14] with yet unsolved reason;
- The discovery of the “uncoupling” of the aforementioned reactions in the presence of N${}^{4}$-OH-dCMP (N4) [15] and disappearing of the cofactor methylene group, still waiting to be explained [16].

None of the above discoveries would be possible without technological developments and improvements in experimental and computational methodologies (especially in crystallography, kinetic isotope effects and quantum mechanical calculations) that help to unravel crucial details. One of the frequently utilised methods is fluorescence spectroscopy, which can be very useful to examine inter- and intramolecular interactions, association processes, denaturation and many others. Fluorescence has the advantage of being sensitive, while being non-invasive and easy to use if molecules (such as TS) have intrinsic fluorescent properties.

More advanced methods such as ARMES (Anisotropy Resolved Multi-Dimensional Emission Spectroscopy) or EEFL (Excitation Emission Fluorescence Lifetime) can be useful in fluorescence analysis of complex, multi-fluorophore proteins, as they span the complete emission spectra [17,18,19,20]. These techniques are particularly useful in the case of tryptophan (Trp), which exhibits strong dependence of the emission properties on its environment; however, interpretation of its fluorescence spectrum and lifetime is not straightforward [21,22,23].

**Figure 1:**
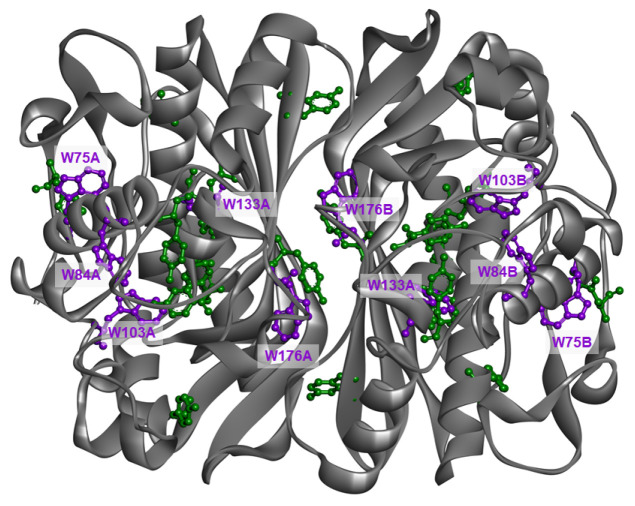
mTS with marked Tyr (green) and Trp (purple) residues. PDB ID: 3IHI.

The first aim of this work is to report new spectroscopic data related to mTS and also present the first spectroscopic data about its two mutants (H190A and W103G). In these mutants, histidine at position 190 and tryptophan at position 103 are substituted with alanine and glycine, respectively. The substituted amino acids were chosen intentionally, as they are thought to play important roles in catalysis and substrate binding. The second important objective was to examine binary complexes of the WT mTS enzyme and its mutants with the substrate (dUMP) and two inhibitors, FdUMP (the active form of several drugs used in cancer therapies) and N${}^{4}$-OH-dCMP (N4) [24,25,26,27]. Studying these interactions is important as it may shed more light on the details of the mTS reaction, which in turn might help in, e.g., cancer treatment improvement. As the examined enzymes have multiple fluorophores (mTS has ten Trp residues and 22 Tyr residues, Figure 1), described goals were obtained by examination of their intrinsic fluorescence using multi-dimensional, steady-state and time-resolved spectroscopy techniques, reinforced with in silico calculations.

## 2. Results

### 2.1. Fluorescence Spectra

**Figure 2:**
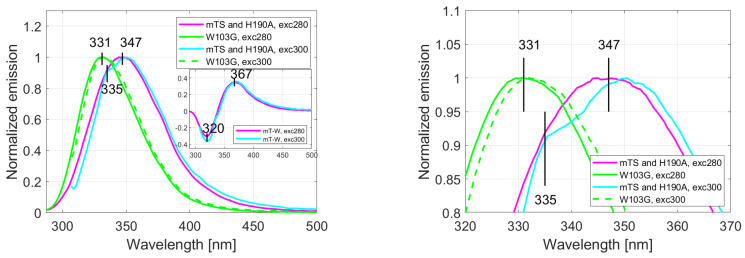
(**a**) Emission spectra of mTS, H190A and W103G recorded with excitation at $\lambda_{exc}$ = 280 nm and 300 nm. Inset: difference spectra calculated after normalisation of mTS and W103G spectra recorded with excitation at $\lambda_{exc}$ = 280 nm (magenta) or $\lambda_{exc}$ = 300 nm (cyan). (**b**) Magnification of the WT mTS enzyme and its mutants emission spectra with a close view of their maxima.

The fluorescence spectra of H190A and recombinant mTS recorded with excitation at $\lambda_{exc}$= 280 nm and 300 nm were identical, with a common emission maximum at 347 nm. However, upon excitation at $\lambda_{exc}$ = 300 nm, a shoulder appears at 335 nm (Figure 2a,b). This shoulder matches the W103G mutant emission maximum, which is surprisingly strongly blue-shifted relative to the mTS/H190A, and is located at 331 nm (independently of $\lambda_{exc}$). The long-wavelength tail emission observed for mTS/H190A is also strongly quenched in W103G. The difference spectra obtained between mTS/H190A and W103G are shown in Figure 2a, where a minimum at 320 nm and a maximum at 367 nm are observed. These findings prompted us to investigate if mTS fluorescence is multi-component and to determine the contribution of Trp103. It is worth mentioning that in spite of the differences seen in their fluorescence spectra, the WT mTS enzyme and its mutants have the absorption maxima located at the same wavelength, λ = 282 nm (not shown).

### 2.2. MDF and Chemometrics

**Figure 3:**
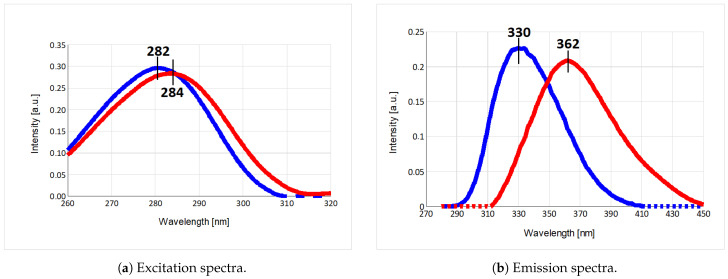
Excitation and emission spectra of mTS emission components calculated from the model based on all of the measured samples with help of the PARAFAC (Section 4.5; (**a**) excitation spectra and (**b**) emission spectra).

**Figure 4:**
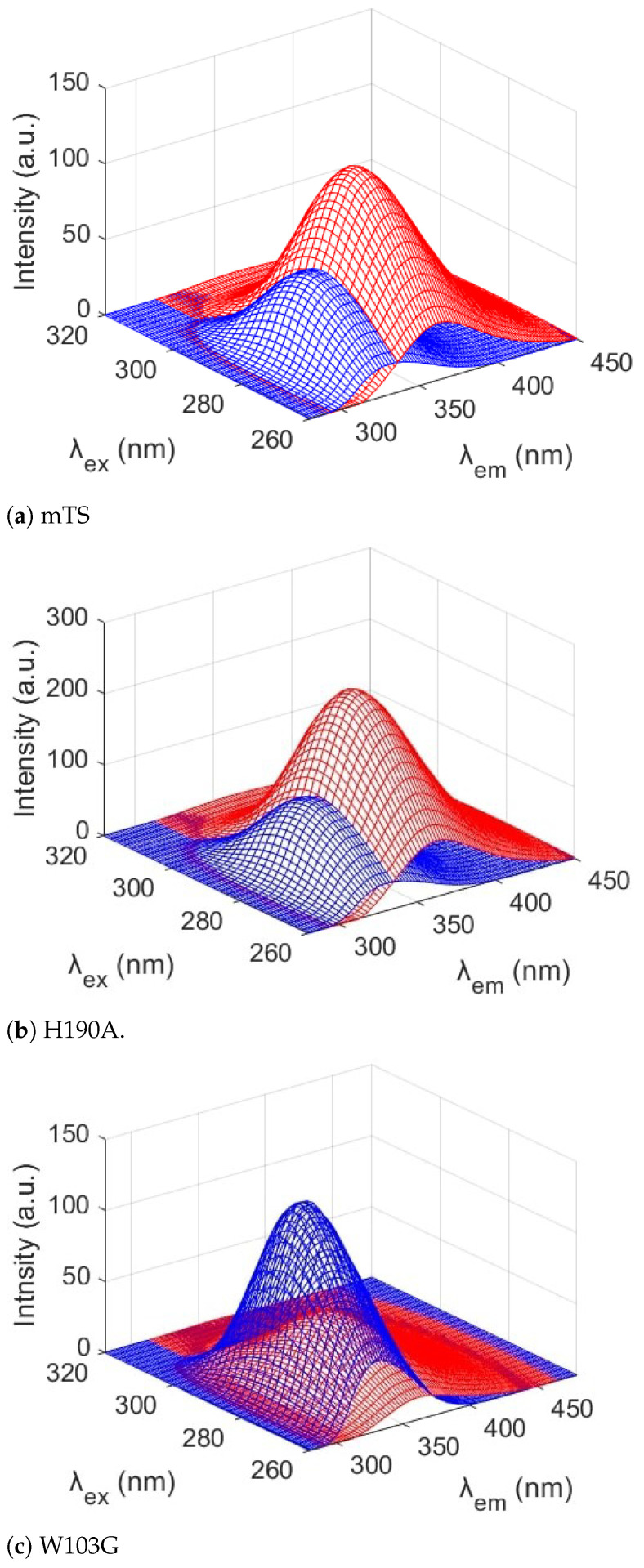
Emission components resolved using PARAFAC analysis for (**a**) mTS, (**b**), H190A, and (**c**) W103G. $\lambda_{ex}$—excitation wavelength, $\lambda_{ex}$—emission wavelength.

Application of multi-dimensional fluorescence (MDF) with PARAFAC data analysis confirmed our hypothesis that mTS fluorescence was the result of different Trp emitters. The excitation and emission spectra of the resolved components (Figure 3) and the MDF spectra resolved for all examined enzymes (Figure 4) correlate with the results described in the previous section. To determine the validity of the model for two components presented here, a diagnostic tool CONCORDIA was used (see Appendix A, Table A1 and Table A2 [28]).

The PARAFAC analysis of mTS emission strongly suggests the presence of two major fluorescence components, showing almost identical maxima of excitation (λ = 282/284 nm, Figure 3a) but substantially separated emission maxima (λ = 330/362 nm, Figure 3b). The 330 nm maximum correlates with the W103G emission maximum and the shoulder seen at 335 nm in the mTS and H190A emission spectra recorded for $\lambda_{exc}$ = 300 nm (Figure 2, Section 2.1). The 362 nm maximum resembles the maximum of the mTS-W103G difference spectra (inset in Figure 2a), which suggests that it could be identified as the Trp103 emission maximum.

The latter indicates that Trp103 is more solvent accessible, which contradicts the results of solvent exposure calculations of the Trp residues in mTS, suggesting their solvent inaccessibility (cf. Section 4.8). Moreover, Trp103 seems to be almost solely responsible for the long-wavelength emission and the fluorescence red-shift, because its absence in the W103G mutant (Figure 4c) substantially decreases the intensity of the long-wavelength component (represented by the red colour in Figure 3 and Figure 4) when compared to the mTS and H190A (Figure 4a,b, respectively). Thus, Trp103 emission should be identified with the component of the mTS emission marked in red (the maximum at $\lambda_{max}$ = 362 nm), whereas the other tryptophan residues seem to be responsible for the “remaining” emission ($\lambda_{max}$ = 330 nm), Figure 3b and Figure 4.

One should also notice, that the maximum of the “red component” is also red-shifted in respect to the emission maximum of a Trp residue in a highly polar environment such as ethanol [29]. Therefore, we hypothesise that, even though the Trp103 side chain is directed toward the protein interior (cf. GETAREA [30] results), its local environment’s electrostatic potential differs from that of the rest of Trp residues and thus is responsible for Trp103’s unique spectral properties [22,31].

### 2.3. Electrostatic Potential

**Figure 5:**
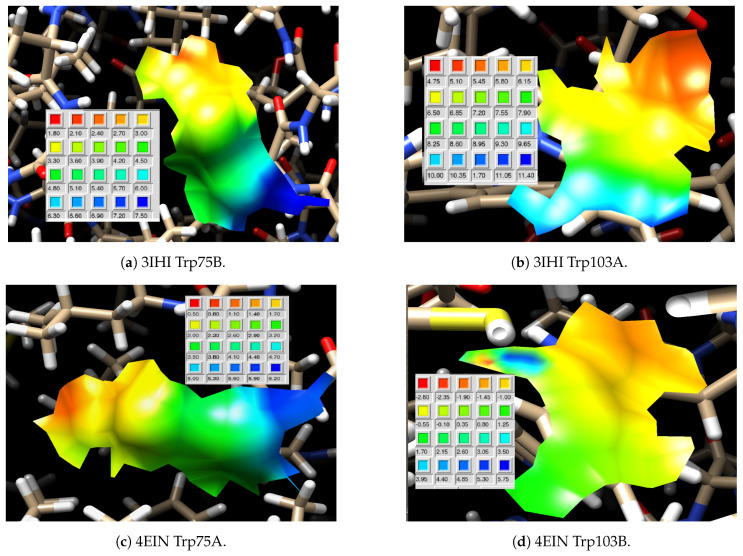
Local electrostatic potential of (**a**) Trp75, chain B and (**b**) Trp 103, chain A from mTS (PDB ID: 3IHI) and (**c**) Trp75, chain B and (**d**) Trp 103, chain A from mTS + N^4^-OH-dCMP (PDB ID: 4EIN). Colour scales for the electrostatic potential are selected to fit the individual ranges of Trp residues. Colours indicate the local values of electrostatic potential.

**Figure A1:**
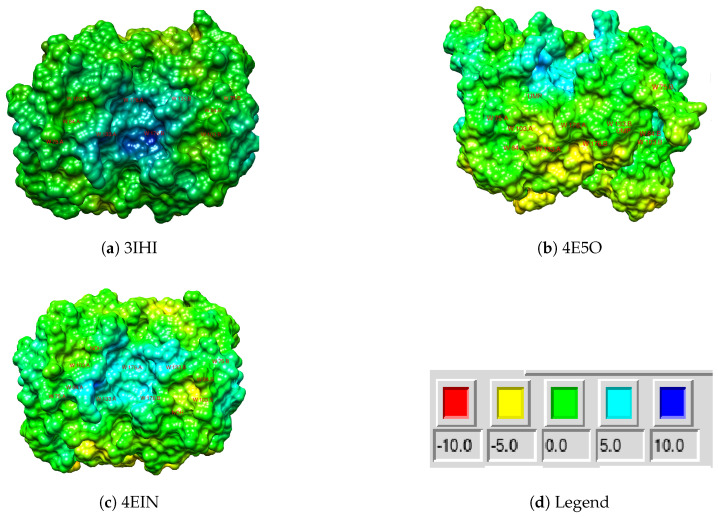
Surface electrostatic potentials of (**a**) mTS (PDB ID: 3IHI), (**b**) mTS + dUMP (PDB ID: 4E5O) and (**c**) mTS + N4 (PDB ID: 4EIN) structures. For better clarity, the narrowed range of potential from −10 to +10 is shown in arbitrary units. Colours (**d**) indicate the local values of electrostatic potentials.

To verify the hypothesis about the presence of the two emission components (of which, one is mainly Trp103 fluorescence) that could originate from differences in the electrostatic potentials (E.P.) in the local environments of Trp residues, the APBS computational methodology was used to calculate the E.P. in mTS. Calculations were also performed for two mTS complexes: mTS + dUMP and mTS + N${}^{4}$-OH-dCMP (Appendix A, Figure A1a–c, respectively), to verify the impact of binding with the substrate or inhibitor on the distribution of E.P. over the enzyme. In silico results agreed with the experimental data (Section 2.1 and Section 2.2) and support our hypothesis. Figure 5 presents the results for Trp75 and Trp103 in 3IHI (mTS) and 4EIN (mTS + N${}^{4}$-OH-dCMP) structures, which are examples of the local electrostatic potential around Trp residues.

The E.P. values around the tryptophan residues and their changes are listed in Table 1. The direction of E.P. change is taken arbitrarily along the vector parallel to the long axis of the indole ring and directed from benzene to pyrrole (benzene → pyrrole). In addition, a parameter **Δ** was calculated that shows the difference between the E.P. values at the pyrrole and benzene rings of indole. The minus and plus signs of **Δ** denote how the value of E.P. changes when going from the benzene to the pyrrole ring (“+”: E.P. increases, the electron density decreases; “-”: E.P. decreases, the electron density increases).

The electrostatic potential for each tryptophan residue in mTS (3IHI) is positive, with Trp133 standing out from the others with very high values (though negligible Δ). The lack of considerable differences of E.P. and (mostly) Δ between the subunits of mTS indicates that both subunits of uncomplexed mTS are equivalent. The highest net differences of parameter Δ (around 5) are found for two amino acids with the highest Acc values, Trp75 and Trp103. For most Trp residues, the E.P. values increase from the benzene to the pyrrole ring, except for Trp103. This is probably responsible for the red-shifted Trp103 emission of this amino acid (more details below and in Section 3). This seems surprising, as the solvent accessibility calculated with GETAREA [30] is lower for Trp103 than for Trp75 (Table 1), suggesting a relatively stronger hindrance of Trp103 in the protein interior. Although Trp75 is more solvent exposed and has a similar absolute value of Δ as Trp103, their Δ values have opposite signs. In fact, only Trp103 in both subunits has a negative E.P. change in the benzene → pyrrole direction. Hence, such an E.P. arrangement around Trp103 might be caused by a moderately extensive hydrogen bond network including 3 or 4 hydrogen bonds held by this residue in subunit A or B, respectively, which indicates its role in the active site protection against the solvent [32].

Altogether, fluorescence spectroscopy, ARMES and APBS results show that emission of mTS consists of two major components: (1) with an emission maximum at approx. 330 nm (due to Trp75, Trp84, Trp133 and Trp176) and (2) with a maximum of emission at ca. 362 nm, that results from a single Trp residue, Trp103. According to Burstein et al. [33] and their further works [34,35,36], fluorescence spectra of tryptophan in proteins can be decomposed into emission of individual residues and correlated with parameters of their local microenvironments. Based on emission maxima and designated microenvironment parameters, five Trp classes were suggested: A, S, I, II and III, with A class being buried deepest in the protein interior (with emission maximum at 308 nm, e.g., Azurin) and III class being totally exposed to the solvent, with the maximum of emission in the range of 346–350 nm.

To compare various microenvironments of Trp residues in free mTS and in its complexes with dUMP and N${}^{4}$-OH-dCMP, we have analysed the most reliable parameters according to [36]: the density parameter Den2 (the number of neighbouring atoms less than 7.5 Å from the indole ring) and the residue solvent accessibility (calculated with GETAREA and then roughly adjusted to the Acc parameter, see Section 4.8). Trp residues can be mostly ascribed to S/I classes characterised by an emission maximum between the ranges 321–325 nm and 330–333 nm. The emission maximum of the W103G mutant falls into this range (Figure 2). Trp75, the tryptophan residue most exposed to the solvent, is ascribed to class II (emission maximum in the range of 341–344 nm). Trp103, depending on the parameter, should be ascribed to class I or II. According to these results, correlation between calculated parameters and the *Burstein* Trp classification from blue emission group (all except Trp103) is well established. However, while Acc and Den2 parameters suggest that Trp103 should be buried in the protein interior or only slightly exposed (with the emission maximum between 330 and 344 nm), its strongly red-shifted emission spectrum drastically differs from typical spectra of Trps in this class. Hence, it seems that this residue does not fit into the *Burstein* classification.

The results of calculations for the mTS + dUMP complex (4E5O structure) show that parameters Acc and Den2 proposed in [36] in most cases are not susceptible to the presence of dUMP.

The most pronounced effect is the change of Den2 value for Trp103 from 106 to 128 in one of the two subunits. However, this increase cannot be ascribed with certitude to the presence of dUMP, as the ligand is present and similarly located in both active sites of the structure. Importantly, based on Acc and Den2, after binding of dUMP, Trp103 should be solely classified as Trp of class I (emission maximum in the range of 330–333 nm). The increase of Den2 is also observed for Trp176 in both subunits, however, without changing their classification. On the other hand, considerable changes are observed for E.P. and Δ between the subunits in mTS + dUMP complex. A closer investigation reveals that E.P. values generally decrease after complexation with dUMP, indicating an overall increase in the electron density around the indole rings. The signs of Δ (except Trp84) remain also the same and unchanged compared to free mTS structure (3IHI), emphasizing the role of E.P. in the determination of Trp103 properties that are likely connected to its functionality. The role of E.P. in the functionality of the enzyme has been also demonstrated in ref. [37], in which the distribution of E.P. through the mTS molecules has been proven to be important in both binding of substrates and catalysis.

Considering the Δ values, that clearly show differences between the subunits, the most substantial changes induced by binding with dUMP involve those corresponding to Trp84, Trp103, and to a lesser extent Trp133. Of note are large differences in E.P. values in the vicinity of the latter Trp residue between the subunits (ca. 7 and 20). The difference between Trp103 in both subunits lies in the decrease in the Δ value upon dUMP binding for one of them, while for the second subunit no influence of dUMP is observed. However, whereas the Δ parameter is similar for Trp103 in both subunits of 3IHI (free mTS), for Trp84, which lies in the closest proximity of Trp103, both E.P. and Δ significantly differ between the subunits. In this case, binding dUMP not only causes that the difference (Δ) in E.P. values between the benzene and pyrrole rings of Trp84 becomes comparable with those for Trp103 and Trp75 (the biggest ones) but also the E.P. over the benzene ring becomes negative, corresponding to a concentrated electron density. Of note are also differences in E.P. values between the subunits observed for Trp84, Trp133 and, to a lesser extent, Trp176. Relative to the values calculated for free mTS (3IHI), they are considerably larger. Based on the above results, three important observations can be made: (1) the direction of the E.P. increase is unchanged after dUMP binding and is unique for Trp103; (2) dUMP binding provokes inhomogenous distributions of E.P. between subunits which could be a result of cooperation between them (mTS is known to exhibit negative cooperativity, thus being a half-the-sites active enzyme) [38,39,40]; (3) possibly a strong cooperation between Trp84 and Trp103, which at the closest point are approx. 5 Å away, exists.

Binding of N${}^{4}$-OH-dCMP to the mTS active site (the 4EIN structure) does not influence the Acc and Den2 parameters for Trp75, Trp84 and Trp176. In contrast, for Trp103, both Acc values increase causing the classification change from class I to I/II. Additionally, Den2 in one subunit increases (similarly to the 4E5O structure of the mTS+dUMP complex), which corresponds to the change in the classification from II to I/II. For Trp133, the Den2 parameter is unaffected, however, both Acc values increase by a factor of 2.35 (on average), which changes its classification from S to I. On the other hand, a similar drop of the E.P. values (except for Trp133), as that observed in complexes with dUMP compared to free mTS (3IHI structure), is registered. Two tryptophan residues have negative E.P. over the benzene ring (Trp84A and Trp176B). However, in contrast to the 4E5O structure, no substantial differences between subunits are observed in terms of the E.P. and Δ values. For Trp103, the values of Δ are similar to those for 4E5O structure and show the largest disparity between the subunits. Of note is also an increase in E.P. values for Trp133, which might be connected to the increase in Acc and might follow the reorientation of this residue. Comparison of 4E5O and 4EIN structures (mTS +dUMP and mTS + N${}^{4}$-OH-dCMP, respectively) shows moderate differences in the interactions of mTS with dUMP and N${}^{4}$-OH-dCMP (two additional contacts in 4EIN compared to 4E5O: carbon–hydrogen bond between the imidazole side chain of His190 and N4 atom of N${}^{4}$-OH-dCMP as well as a conventional hydrogen bond between Gln208 and O2 atom of N${}^{4}$-OH-dCMP) and a weaker cooperation between subunits in the mTS + N${}^{4}$-OH-dCMP complexes, reflected by similar E.P. and Δ values for both subunits.

### 2.4. Fluorescence Lifetimes

**Figure 6:**
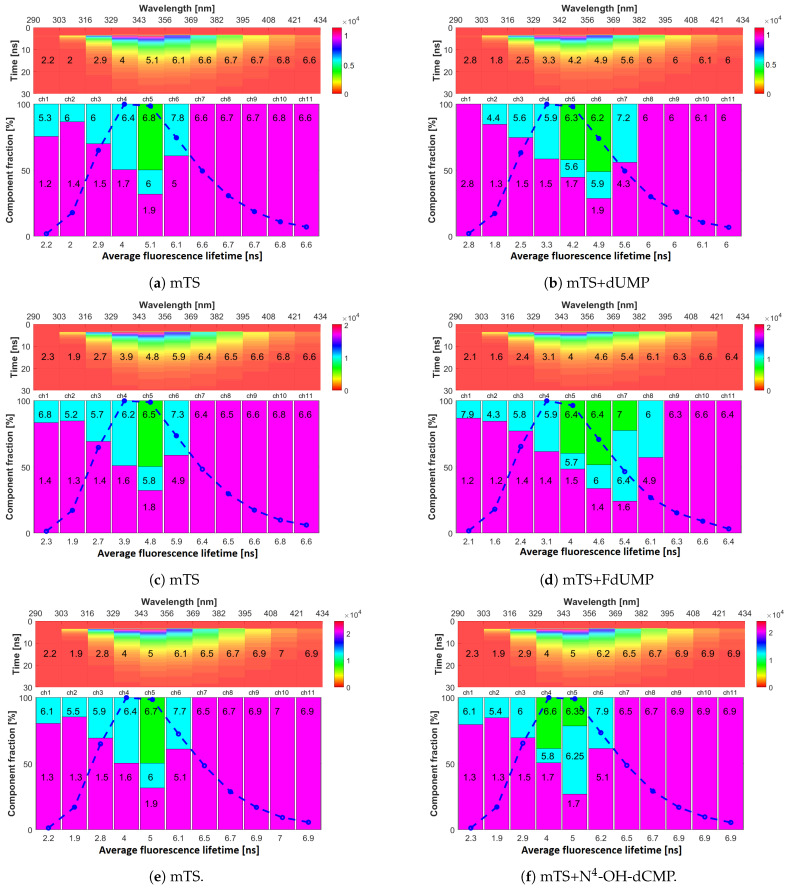
Multichannel time-resolved fluorescence data for mTS (**a**,**c**,**e**) and its complexes with (**b**) dUMP, (**d**) FdUMP and (**f**) N^4^-OH-dCMP. Top panels—vertical axis corresponds to time after excitation and colours reflect fluorescence intensity (photon counts). Spectral ranges of each emission channel are shown on the top horizontal axis, and mean values of fluorescence lifetimes ($\tau_{av}$) are printed on these graphs. Bottom panels—present colour-coded information on decay components of fluorescence decays. Each bar corresponds to one decay component with its height corresponding to its fraction in the fluorescence decay. Decay time $\tau_{n}$ (in nanoseconds) of each component is printed on the corresponding bar. The blue dash-dot line represents the intensity registered in each emission channel and, therefore, reflects the shape of the emission spectrum. Under the bottom panels, average fluorescence lifetimes ($\tau_{av}$) are displayed.

**Figure 7:**
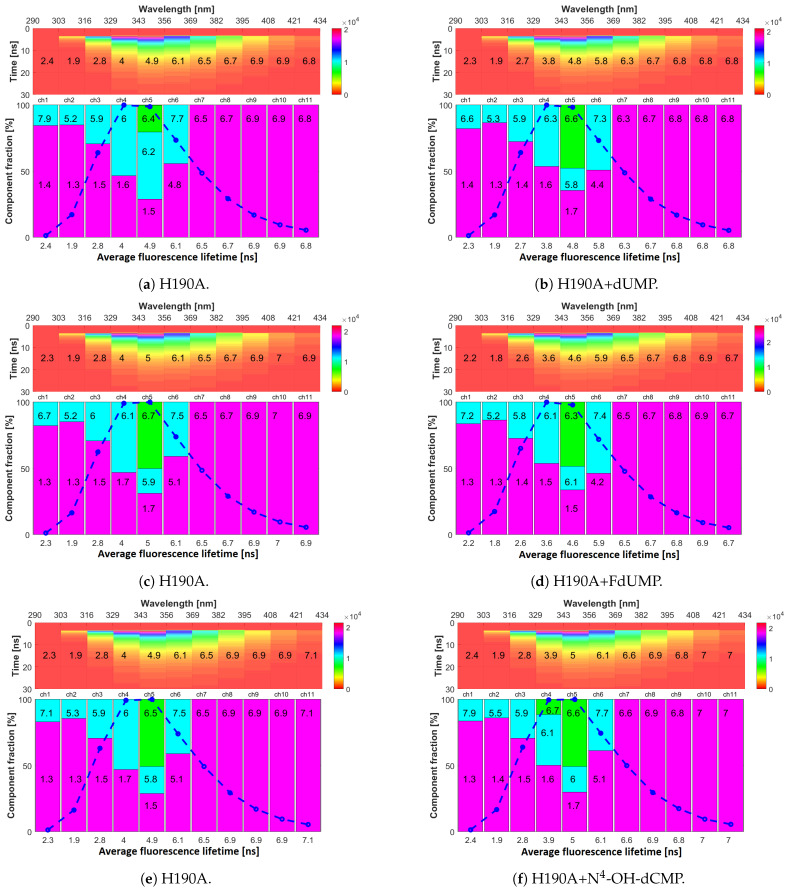
Multichannel time-resolved fluorescence data for H190A (**a**,**c**,**e**) and its complexes with (**b**) dUMP, (**d**) FdUMP and (**f**) N^4^-OH-dCMP. Top panels—vertical axis corresponds to time after excitation and colours reflect fluorescence intensity (photon counts). Spectral ranges of each emission channel are shown on the top horizontal axis, and mean values of fluorescence lifetimes ($\tau_{av}$) are printed on these g raphs. Bottom panels—present colour-coded information on decay components of fluorescence decays. Each bar corresponds to one decay component with its height corresponding to its fraction in the fluorescence decay. Decay time $\tau_{n}$ (in nanoseconds) of each component is printed on the corresponding bar. The blue dash-dot line represents the intensity registered in each emission channel and, therefore, reflects the shape of the emission spectrum. Under the bottom panels, average fluorescence lifetimes ($\tau_{av}$) are displayed.

**Figure 8:**
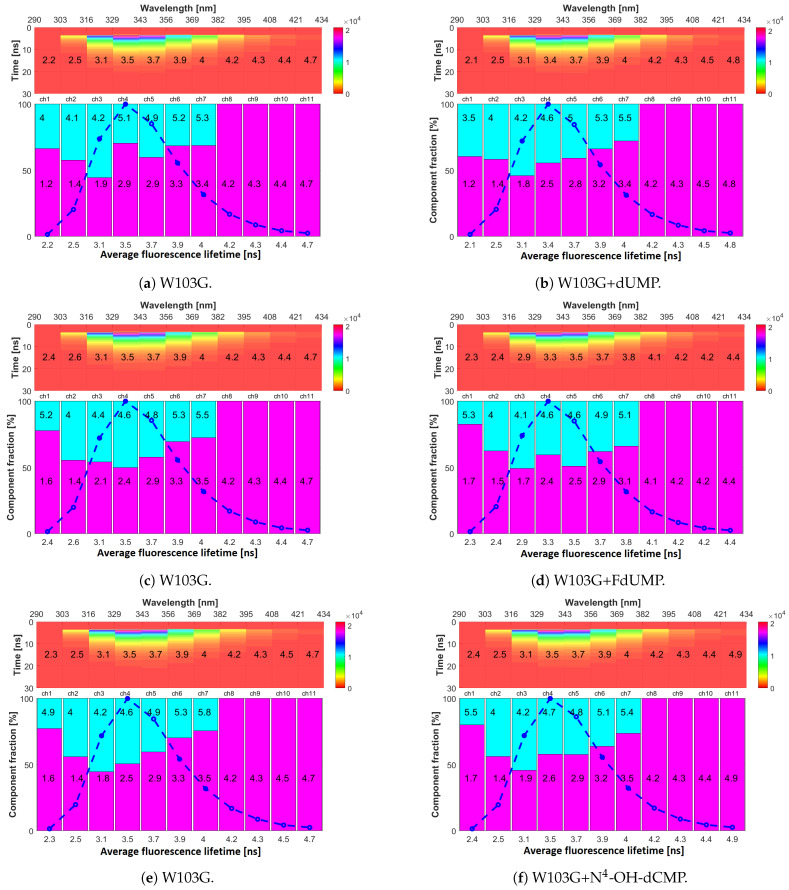
Time, emission wavelength, intensity maps of (**a**,**c**,**e**) W103G and its complexes with (**b**) dUMP, (**d**) FdUMP and (**f**) N^4^-OH-dCMP. Top panels—vertical axis corresponds to time after excitation and colours reflect fluorescence intensity (photon counts). Spectral ranges of each emission channel are shown on the top horizontal axis, and mean values of fluorescence l ifetimes ($\tau_{av}$) are printed on these graphs. Bottom panels—present colour-coded information on decay components of fluorescence decays. Each bar corresponds to one decay component with its height corresponding to its fraction in the fluorescence decay. Decay time $\tau_{n}$ (in nanoseconds) of each component is printed on the corresponding bar. The blue dash-dot line represents the intensity registered in each emission channel and, therefore, reflects the shape of the emission spectrum. Under the bottom panels, average fluorescence lifetimes ($\tau_{av}$) are displayed.

Multi-wavelength time-resolved fluorescence measurements were then used to try to confirm previous results, to find a correlation between E.P. and fluorescence lifetimes, and further characterise the different mutants and their interaction with various nucleotides. The results are shown in Figure 6, Figure 7 and Figure 8 and in Table 2, where top panels represent the fluorescence decays recorded in eleven emission channels, and bottom panels the resolved decay components. Depending on the emission wavelength, between one and three decay components were detected.

**Figure A2:**
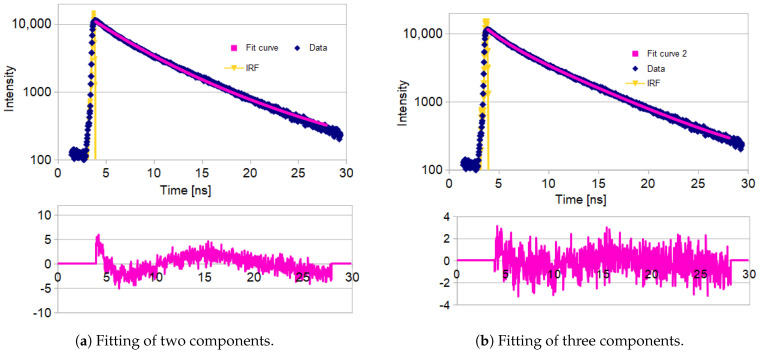
Fluorescence decays registered for channel no. 5 (343–356 nm) for the mTS sample presented in Figure 6a. (**a**) Results obtained after fitting two components, (**b**) results obtained after fitting three components. On the top panels of both sub-figures in blue—registered data as intensity (photon counts), in yellow—IRF. On the bottom panels of both sub-figures, plots of the residuals for the fitted function are presented.

An immediate observation was that the substitution of histidine to alanine (His → Ala) in H190A did not have a substantial impact on fluorescence lifetimes and decay components, as shown in Figure 6 and Figure 7, respectively (left sides). For mTS and H190A, the experimental data showed a monoexponential decay with lifetime of 6–7 ns in 369–434 nm range (channels 7–11), and a biexponential decay with short (<2 ns) and long lifetime (5–6 ns) in 290–369 nm range (channels 1–6). The data collected in channel 5 were fitted considerably better with a three-exponential decay with two long-lived components (5–7 ns), yielding lower $\chi^{2}$ values, and better residuals plots, as shown in Figure A2, Appendix A.

Based on the results presented in Section 2.1 and Section 2.2, the long decay components observed in the red part of the emission spectrum could be attributed to Trp103 (Figure 6a). The second long-lived component, registered in the blue part of the spectrum, most probably originates from emission of other tryptophan residues present in the enzyme as its spectral position and decay time are typical of tryptophans in the protein environment. However, the origin of the short component, seen in the 290–356 nm range, is not so straightforward to determine. It could be attributed to Tyr emission, even if Tyr → Trp FRET is known to often quench it totally [22,23]. Another possibility is that it might originate from electron transfer in the Trp(CE3) → peptide bond (electron acceptor) in direction [41]. If the latter was true, it would mean, that we observe two main types of Trp rotamers in mTS regarding the position in relation to the peptide bond (excluding Trp103).

A substantial difference is observed between mTS and the W103G mutant (Figure 8, left half). Removal of Trp103 results in a considerable decrease in the mean lifetime of fluorescence recorded in the 343–434 nm range (channels 5–11, Table 2). The position of maximum fluorescence in W103G appears also to be blue shifted to 332 nm, with long-wavelength emission intensity being quenched by approx. 15–20% in respect to mTS/H190A. These observations are consistent with the previous attribution of the long-wavelength emission to Trp103 based on steady-state and MDF measurements (Section 2.1 and Section 2.2).

Results of W103G also revealed the presence of only two decay components across all channels. The fraction of long-lived component increase in 290–329 nm range, compared to mTS/H190A, possibly is related to the excited state energy homotransfer between Trp residues in mTS/H190A towards (mostly) Trp103. The non-negligible Trp absorption and the relatively long excited-state lifetime (over 6 ns) of other Trps in 290–315 nm range are long enough for homoFRET to occur in mTS/H190A. Therefore, elimination of Trp103 (W103G) leads to reduction of homoFRET followed by an increase in the long-component fraction.

Results obtained for binary complexes of mTS with dUMP, FdUMP and N${}^{4}$-OH-dCMP (right half of Figure 6) show that values of the mean fluorescence lifetime $\tau_{av}$, and decay times of each component $\tau_{n}$, as well as the pattern of their fractions (*a*${}_{n}$), strongly depend on the ligand.

Binding of dUMP to the active site of mTS (Figure 6b) leads to a decrease in the mean fluorescence lifetime ($\tau_{av}$) in all emission channels compared to unbound mTS (Figure 6a). The strongest fluorescence quenching is observed in the 329–382 nm range (15%–20% reduction of $\tau_{av}$). In general, the lifetime of the short-lived component is unaffected through the whole emission spectrum, whereas shortening of both long components (accompanied by a change of their fractions) is observed. The most pronounced effect of binding with dUMP is the appearance of new fluorescence decay components in the 356–382 nm range (channels 6 and 7, Figure 6b vs. Figure 6a).

Binding of FdUMP, a close dUMP analogue (Figure 6f) results in a decrease in $\tau_{av}$ in all eleven channels, with the stronger quenching effect in the 329–382 nm and weaker in the 408–434 nm range, than for dUMP. A third component also appeared in fitting of channels 6 and 7 (Figure 6d).

Binding of N${}^{4}$-OH-dCMP did not impact $\tau_{av}$ (Figure 6f). The only noticeable aspect was the appearance of an additional long component (t = 5.8 ns) in the 329–343 nm range (channel 4) which might be a result of the increase in E.P. value at the position of Trp133 mentioned in Section 2.2. Different reactions on N${}^{4}$-OH-dCMP binding are observed for long components in the 343–358 nm range (channel 5). Although a three-exponential fit was much better than two-exponential ( $\chi^{2}$ = 1.5 and $\chi^{2}$ = 5.4, respectively), the long decay times (6.35 and 6.25 ns) are too similar to make any conclusion. One should probably expect a bi-exponential fit if, e.g., a model based on continuous distribution of fluorescence lifetimes [42] was used instead of multi-exponential decay.

In the case of H190A (Figure 7, right half), it was clear that the binding of all ligands (dUMP, FdUMP and N${}^{4}$-OH-dCMP) had a smaller impact on $\tau_{av}$ and relative decay components contributions compared to mTS complexes, even though based on initial fluorescence quenching data (not shown), the complexes formed by H190A with dUMP/FdUMP appeared only slightly weaker or comparable to mTS ones.

Upon binding with dUMP, for example (Figure 7b), a very slight reduction at most could be observed in decay times of both long components registered in 329–382 nm range. This leads to almost no change in $\tau_{av}$, illustrating the lesser influence of dUMP on H190A fluorescence properties compared to mTS. The same was seen for H190A with N${}^{4}$-OH-dCMP (Figure 7f). Only the binding to FdUMP seemed to have a slightly stronger influence (Figure 7d) on H190A fluorescence properties, as $\tau_{av}$ decreased a bit more in the 316–369 nm range.

Taking together results for dUMP and FdUMP, it appears that H190A fluorescence decay behaviour is independent of the fluorine presence, suggesting that His190 might play an important role not in the binding itself, but in the inter- and intra-molecular interactions based on E.P. distributions and as a mediator between ligands and fluorophores.

On the other hand, TSCPC, together with results presented in Section 2.5 and Appendix A for N${}^{4}$-OH-dCMP, suggests rather different conclusion (see further herein).

For W103G, the binding to dUMP, FdUMP and N${}^{4}$-OH-dCMP (Figure 8, right panels) did not seem to impact $\tau_{av}$. Only small changes in individual components’ decay times were observed, which were rather unsubstantial, random and inconclusive. This suggested lack or very weak interactions and therefore emphasises the role of Trp103 in nucleotides binding.

### 2.5. Interaction of His190 with N${}^{4}$-OH-dCMP

**Figure 9:**
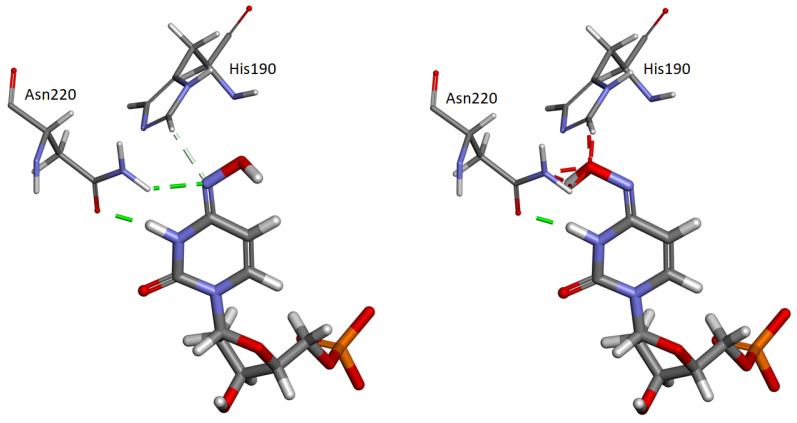
Interaction of anti-imino (**left**) and syn-imino (**right**) N${}^{4}$-OH-dCMP with Asn220 and His190 amino acid residues in the mTS active center (based on crystal structure 4EIN [16]). Dashed lines represent hydrogen bonds, carbon hydrogen bonds according to [46], and bumps (green, light green, and red, respectively). Prepared using Receptor–Ligand Non-bond Interactions tool in Discovery Studio Visualizer (Dassault Systemes, Velizy-Villacoublay, France).

Analyses of crystal structures of mTS bound with N${}^{4}$-OH-dCMP suggest that the molecular mechanism of an apparent strong preference for the anti rotamer of the imino inhibitor form, described in [43], is connected with the His190 presence. It was confirmed by both crystallographic studies [16,44] and molecular dynamics simulations [27] that the presence of His190 causes a steric hindrance preventing the *syn* form from being bound (Figure 9).

**Figure A3:**
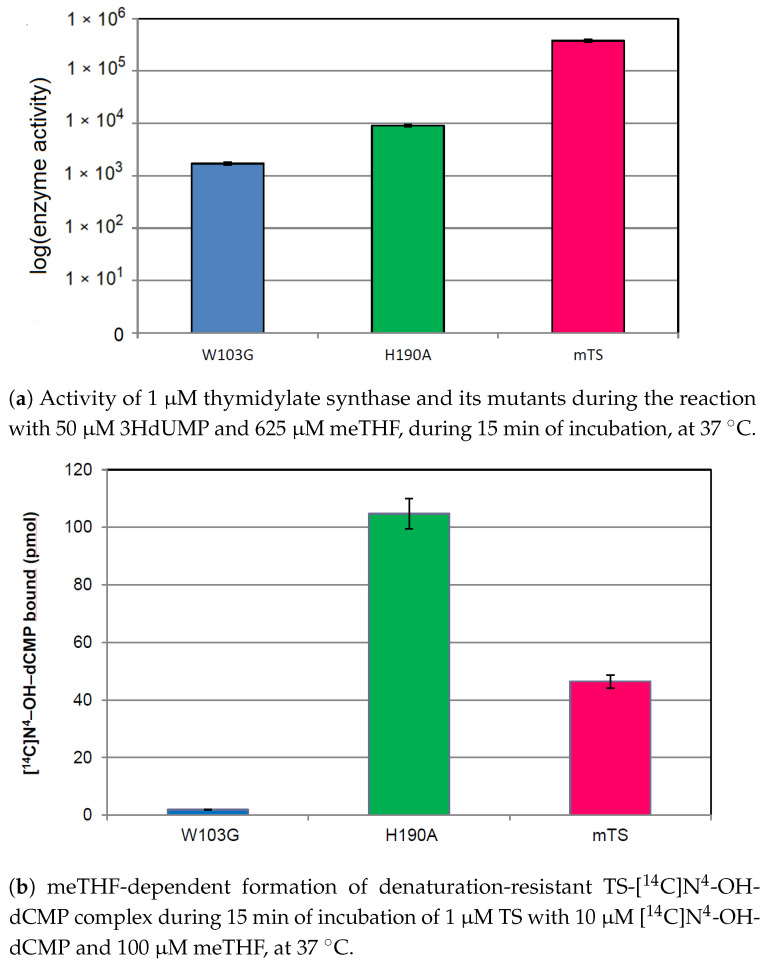
(**a**) Logarithm of activity in [pmol × 10^−2^ × min^−1^ × mL^−2^] of thymidylate synthase and its mutants and (**b**) meTHF-dependent formation of denaturation-resistant TS-[^14^C]N^4^-OH-dCMP complex.

In search of an experimental support for the latter hypothesis, mTS and its two mutant forms, H190A and W103G, were compared with regard to the specific activity and capacity to bind the inhibitor. Albeit both mutant enzymes, H190A and W103G, compared to the unaltered enzyme, showed much lower specific activities (Appendix A, Figure A3), comparison of their abilities to bind N${}^{4}$-OH-dCMP in the reaction with mTHF (determined by monitoring a denaturation-resistant complex formation [15]), presented surprisingly different profiles. While with the W103G mutant the binding capacity, compared with the enzyme activity, underwent much stronger reduction in comparison to the unaltered enzyme, the H190A mutant showed twofold higher ability to bind the inhibitor than the unaltered mTS (Appendix A Figure A3b). Interestingly, the H190A mutation had no influence on the apparent K${}_{m}$ values for dUMP and mTHF [45]. Considering a 10-fold lower specific activity of the H190A form vs. unaltered mTS, the efficiency of the former to catalyse mTHF-dependent binding of N${}^{4}$-OH-dCMP is approx. 20-fold higher than that of the latter. In this view, an apparent lack of change of the fluorescence lifetime of H190A in the complex with N${}^{4}$-OH-dCMP, might in fact represent strong static quenching.

## 3. Discussion

Spectral properties of tryptophan, such as the position of the emission maximum, the fluorescence quantum yield and the character of the fluorescence decay, strongly depend on its local environment [21,22,23], allowing one to study structural and dynamic properties of proteins using fluorescence spectroscopy. Unfortunately, the relationships between spectroscopic features of tryptophan and properties of its environment are very complex and difficult to categorise. For example, many attempts to explain the non mono-exponential decay of the tryptophan fluorescence have been made and several hypotheses were proposed, the most commonly accepted being: (1) the rotamer hypothesis (multiplicity of conformational states of the residue in the ground state) [41,47,48,49,50]; (2) the charge/electron transfer hypothesis (excited state reactions); (3) distribution of the local electric field intensity and direction [51,52]; (4) spectral relaxation [53]. *Hudson* [54] proposed also that the bi-exponential decay may originate from ionisation of an excited state of tryptophan (by the electron transfer to an adjacent residue), followed by formation of an ion-pair complex with a unique lifetime and a rate constant for the return to the original excited state of tryptophan.

A common core of those hypotheses is that regardless of the assumed origin of the spectral shifts and the multiexponential fluorescence decay, they all lead to the excited state quenching by the electron transfer (ET) from the indole ring of Trp to the electron acceptor, in many cases an amide of the protein backbone. On the other hand, the spectral relaxation hypothesis states that as the protein interior acts as a viscous solvent, its response to the transition dipole change following the optical excitation is slower than that of, e.g., water. Hence, the observed behaviour of fluorescence reflects the dynamics of the tryptophan’s environment relaxation. One also has to keep in mind that a protein in a solvent has a flexible form, thus its structure can change in time. Consequently, the energy of the local ground or excited state of Trp residues can fluctuate. In this study, we have assumed that a combination of the rotamer and electric field mechanisms (both with specific ET probabilities) are responsible for the spectral properties of mTS.

In order to better understand the results presented in this work, it is necessary to explain the role of the electrostatic potential and its effect on Trp emission properties, as it will be the basis of the further analysis. From the results of the steady state and time-resolved fluorescence spectroscopy and E.P. calculations, we can infer that the tryptophan at position 103 not only has unique properties but also affects properties and possibly conformations of other tryptophan residues. As mentioned in Section 2.4, homoFRET is most probably responsible for the reduction in the long-lived component’s fraction of the fluorescence decay at the shortest emission wavelengths (mTS and H190A). One could argue that if homoFRET was the reason for the change in emitting component contribution, the long component seen in W103G fluorescence decays (in ch. 1–4) should not be shorter relative to mTS/H190A. However, one has to keep in mind, that with the elimination of Trp103, the conformation of the backbone might be changed and this could influence the fluorescence lifetimes. Of note is that the change of the Trp dipole orientation from destabilizing to stabilizing is around 2 kcal/mol, which is approx. 10% of the Gibbs free energy change due to the protein folding [52]. Besides, the rate of the electron transfer (ET), which we assume is the source of the multiexponential fluorescence decay, depends exponentially on the distance between the C3 atom of the indole ring (electron donor) and the carbonyl C atom of the peptide bond (electron acceptor) (Equation (1)):

(1) $$k_{ET}=k_{0}exp\left(-\beta \left(R-R_{0}\right)\right)$$

where $k_{ET}$ is the rate of the electron transfer, $k_{0}$ is the rate of the electron transfer at the van der Waals distance ($R_{0}$), *R* is the distance between the mentioned carbon atoms, and β is the range parameter that depends on the medium [41,49]. Thus, for a large enough value of the β parameter, a 0.5 Å increase in separation may increase the fluorescence lifetime τ ($\tau^{-1}=k_{r}+k_{nr}$) by a factor exceeding 2, assuming that $k_{r}$ is constant (in the above expression for $\tau^{-1}$, $k_{r}$ is the radiative rate constant and $k_{nr}$ is the non-radiative rate constant that includes contributions from several processes, such as the intersystem crossing ($k_{isc}$), solvent quenching ($k_{sq}$), proton ($k_{pt}$) or electron transfer ($k_{ET}$): $k_{nr}=k_{isc}+k_{sq}+k_{pt}+k_{ET}$).

**Figure 10:**
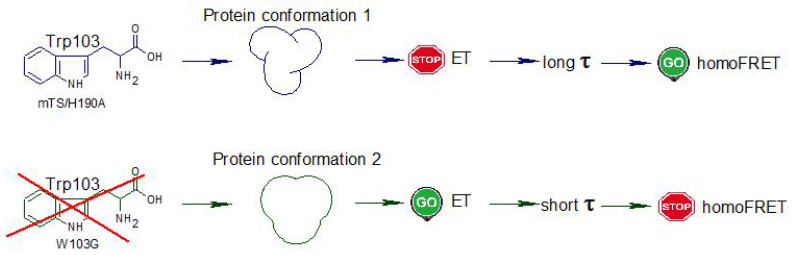
The alleged path of events induced by the elimination of Trp103.

**Figure 11:**
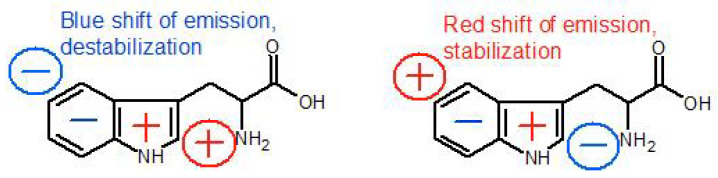
The influence of the electric field on charge distribution in a tryptophan molecule. Figure created based on [51,52].

**Figure 12:**
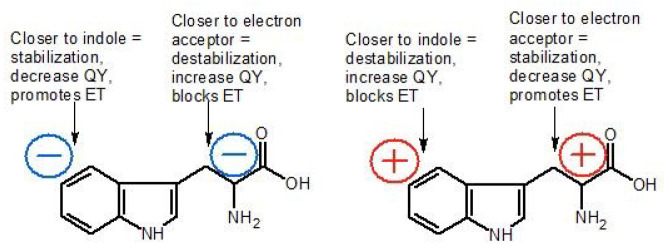
Role of the electric field in ET promoting with the peptide bond as an alleged electron acceptor.

Consequently, the presence/absence of Trp103 might change the rotameric form or orientation of other Trps in such a way that the rate of ET to the backbone amide is significantly modified. If so, in mTS/H190A, the overall protein conformation hinders ET from the tryptophan residues belonging to blue emission group (blue component in Figure 3, all Trp residues except Trp103, slower decay of the long component), whose emission is then quenched through homoFRET. In favour of this hypothesis is also the fact that the energy transfer efficiency is proportional to the donor’s excited-state lifetime (here, the Trps from the blue emission group). Figure 10 presents the above described path of possible events induced by the elimination (substitution) of Trp103. It is also important to note that the decay of the emission in the long-wavelength part of the fluorescence spectrum ($\lambda_{em}$ > 370 nm), ascribed to Trp103, is quite long and apparently consists of only one component. This fact suggests that the direction of the electric field vector at the position of Trp103 blocks ET from the indol ring (Figure 11 and Figure 12).

The second characteristic feature of Trp103 is the large red-shift of its emission (rarely observed) in spite of its location in the protein interior. Such a shift may result from its interactions with the strongly polar environment because it is known that the net effect of polar molecules (including water) on Trp emission can be as large as to cause a shift of its maximum by 60 nm (in respect to the vacuum emission of Trp) [52,55].

The difference between the values of the local electrostatic potential at the benzene and pyrrole rings of Trp103, calculated with APBS (Figure 5, Table 1), is considerable, compared to other tryptophan residues. More importantly, the arrangement of E.P. in respect to the indole’s long axis promotes stabilisation of the excited state (Figure 11), which results in a shift of the emission spectrum towards longer wavelengths (Figure 2, Figure 3 and Figure 4), probably blocks ET and results in the long fluorescence lifetime. The key role of electrostatic interactions in TS substrate binding and catalysis was also suggested in the past [37], however, earlier calculations were focused on the wide surface range (e.g., binding sites), not on specific residues, as in the present study. Moreover, the arrangement of relative values of E.P at the benzene and pyrrole rings of Trp103 does not change during ligand binding (Table 1), which strongly suggests its functional role in either binding of substrates or catalysis (elimination of Trp103 results in a dramatic loss of the mTS activity, not published).

It has to be emphasised that the presence/absence of Trp103 might also influence the E.P./electric field “seen” by the rest of tryptophan residues and cooperate with the rotamers orientations, jointly influencing the mTS spectral properties (Figure 1 and Figure 12).

**Quenching by ligands**

**mTS complexes**

Asymmetric binding of nucleotides has been reported [43,56,57] (dUMP, FdUMP and N${}^{4}$-OH-dCMP) to thymidylate synthases from various sources, or binding (with similar affinities) of only one dUMP or FdUMP molecule per human TS (hTS) dimer [14], an enzyme almost identical to mTS. Reilly et al. [58] showed FdUMP binding to hTS to be asymmetric, involving one high affinity binding site, responsible for stoichiometry of one FdUMP molecule per hTS dimer, and one low affinity binding site—the resulting effective stoichiometry being 1.7 FdUMP molecules per hTS dimer. On the other hand, the stoichiometry of N${}^{4}$-OH-dCMP binding by *L. casei* TS dimer was 1.9 [56,59], suggesting almost identical affinity of both sites, although with a biphasic dissociation. For dUMP, the stoichiometry was reported to be within the range of 1.25–1.6 molecules per TS dimer [56]. Furthermore, a biphasic time-dependence of the inhibitor-dependent inactivation of various TSs was observed during the enzyme incubation with a cofactor and a substrate (dUMP) or its analogues (e.g., N${}^{4}$-OH-dCMP or FdUMP). These observations can be understood in terms of a negative cooperativity between the two subunits [43,60,61,62]. Nevertheless, a recent report [63] showed that for a recombinant mTS, time- and inhibitor-dependent inactivation relationship is linear (for both N${}^{4}$-OH-dCMP and FdUMP), in contrast to the wild-type enzymes from *Mus musculus*, thus attesting to the lack of the cooperativity. This changed, for both types of inhibitors, when the recombinant mTS had been phosphorylated. Hence, it was hypothesised that the biphasic inactivation is a result of the post-translational modifications and thus might be observed only for wild-type TSs. Nonetheless, this conclusion is questionable because the biphasic mode of association occurred also between the recombinant hTS and FdUMP [58]. Notably, from the literature cited above, only Felder et al. [14] investigated binary complexes. Their calculations of dissociation constants of dUMP and FdUMP and the stoichiometry of the dUMP binding were nearly identical with the previous reports in [64,65,66,67], in which binary complexes were also investigated and where the stoichiometry of the dUMP binding was determined to be 1.1 per an enzyme dimer (one active site adopted by dUMP). Thus, in view of the mentioned reports, we can conclude that in the current study of mTS binary complexes, FdUMP and dUMP, as close analogues, bind with a similar affinity and, within most mTS molecules, to the one subunit, while N${}^{4}$-OH-dCMP seems not to have such a strong tendency for asymmetric binding.

The in silico and TCSPC results presented here show the usefulness of these techniques in distinguishing interactions with different types of nucleosides. The binary complexes with dUMP and FdUMP exhibit similar fluorescence decays. The presence of these ligands affects mainly the emission above 340 nm. An additional decay component detected in channel nos. 6 to 8 might be the effect of quenching of the excited state of Trp103, possibly by the electron transfer that follows the stabilizing rearrangements for the electron transfer state between the indole of Trp103 and an electron acceptor (or a change in the rotamer orientation that promotes ET). The incorporation of an additional charge by the fluorine in FdUMP might stabilise electronic transitions to a higher extent, by decreasing the energy gap between the excited (${}^{1}$L${}_{a}$) and the ET state [52]. Further, this promotes the electron transfer, which results in the appearance of the additional component. The result is the stronger quenching of the mTS emission in respect to the mTS+dUMP complexes. Moreover, it was shown [68] that fluoro substitution on the indole ring has a critical impact on the electron transfer and the multiexponential properties of Trp fluorescence decays. A fluorine presence/influence (FdUMP) and the response of the local electrostatic potential in the protein on the biding of a ligand could explain the earlier reported enhanced emission quenching of human TS by FdUMP relative to *E. coli* TS, even though a similar association occurs [14]. The response of the electrostatic potentials in both enzymes on the FdUMP might vary and cause differences in the emission quenching. It was shown by Garg et al. [37], that, e.g., either increase or decrease in *E. coli* TS activity was observed, depending on the localisation of the changes in polarisation.

In contrast, interactions of mTS with N${}^{4}$-OH-dCMP seem not to depend so strongly on the E.P. Moreover, the in silico results indicate that the intercommunication of the subunits (cooperation) is not as pronounced as in the binary complexes with dUMP (Table 1). The results of time-resolved fluorescence measurements also favour this hypothesis: the impact of N${}^{4}$-OH-dCMP on the fluorescence lifetimes distribution is negligible when compared to the complexes with FdUMP and dUMP. The only considerable change, an additional decay component which appears in channel 4, might be linked to the increase in the Acc and Den2 parameters for Trp133. These parameter changes suggest a relationship between the Trp133 rotamer orientation change and the increase in E.P. close to the peptide bond, which would eventually promote the electron transfer. The latter process results in the quenching of the Trp133 emission, accompanied by an appearance of an additional decay component.

**H190A complexes**

It was previously shown that the substitution of the active site histidine in *E. coli* TS (His147 in *Ec*TS, the equivalent of His190 in mTS) [69,70] leads to a decrease in the catalytic capabilities of the enzyme, however, without any effect on K${}_{m}$ of dUMP and mTHF [69]. This observation indicates that His147 in *Ec*TS is not significantly involved in the initial substrate binding. Furthermore, a decrease in the activity is shown for the H190A mutant in respect to the mTS (Figure A3a), which is not accompanied by a substantial influence on K${}_{m}$ for dUMP or mTHF (not shown). However, fluorescence lifetime measurements (Section 2.4) show a major change upon replacement of His190 to Ala190 in complexes with dUMP and FdUMP. This suggests an important role of His190 in proper E.P. distribution necessary for the reaction to occur, considering the above outlined link between E.P. distribution and fluorescence lifetime kinetics (Section 2.3 and Section 2.4 and further explanation herein).

On the other hand, the results presented in the Appendix A Figure A3b, unambiguously show an enhanced binding of N${}^{4}$-OH-dCMP to the H190A mutant (accompanied by the presence of the cofactor) compared to the complexes with mTS, suggesting a marked K${}_{m}$ decrease. In this view, the fluorescence lifetime results obtained for mTS + N${}^{4}$-OH-dCMP might not be so obvious. As mentioned before, the presence of His190 probably forces binding of only the anti-imino form of N${}^{4}$-OH-dCMP, which constitutes only *ca.* 5% of the molecules in solution. However, for the H190A mutant, the absence of His190 probably results in binding of the other rotamer. Therefore, it is possible that the decrease in the N${}^{4}$-OH-dCMP influence on the fluorescence decay (in 329–343 nm range) in the H190A + N${}^{4}$-OH-dCMP complexes, might represent the increase in the binding of the syn-imino form to the H190A and the change of the interactions mode.

**W103G complexes**

It was previously shown that lack of a tryptophan residue in the binding pocket (e.g., Trp80 in *Lactobacillus casei*) is responsible for activity loss [32,71]. Results presented in the Appendix A Figure A3a confirms that this is also true for mouse TS. However, its role was not investigated in detail. Therefore, investigation of the W103G mutant was of big interest in order to prepare comprehensive and detailed work on the spectroscopic features of mTS and their relation to its activity.

The lack of substantial differences is observed between complexes of dUMP, FdUMP and N${}^{4}$-OH-dCMP with W103G. However, it has to be pointed out that this mutant is relatively unstable under continuous optical excitation. Hence, based on the presented results (minimal change in fluorescence lifetimes), the fact that this mutant has a very low activity and lack of mTHF-dependent formation of the stable complex between W103G and N${}^{4}$-OH-dCMP (Appendix A, Figure A3b) allows us to assume an important role of Trp103 in mTS binding with nucleotides. In *Ec*TS, this tryptophan residue is responsible for interactions with HB network and “closing” of the active site. Perhaps Trp103 “armed” with its local electrostatic potential facilitates the nucleotide binding via coulombic interactions.

## 4. Materials and Methods

### 4.1. Materials

(6S)-5,6,7,8-Tetrahydrofolic acid (THF), (6RS)-methylene-5,6,7,8-tetrahydrofolic (mTHF) acid and 7,8-dihydrofolic (DHF) acid were from Schircks Laboratories (Bauma, Switzerland). 5${}^{\prime }$-monophosphate-2${}^{\prime }$-deoxyurydilate (dUMP), 5-fluoro-5${}^{\prime }$-monophosphate-2${}^{\prime }$-deoxyurydilate (FdMUP), hydroxylamine, dCMP, glycerol, lysosyme, Na${}_{3}$VO${}_{4}$, NaF, Na${}_{4}$P${}_{2}$O${}_{7}$, EGTA, streptomycin sulphate, (NH${}_{4}$)${}_{2}$SO${}_{4}$, BSA, Al(OH)${}_{3}$, NiSO${}_{4}$, NaH${}_{2}$PO${}_{4}$, K${}_{2}$HPO${}_{4}$, were products of Sigma-Aldrich (MERCK, Darmstadt, Germany). Methanol, 2-propanol and 1,4-dioksan were products of Chempur (Piekary ląskie, Poland). β-mercaptoethanol (EtSH), KCl, NaCl, pepton, yeast extract, glucose, ampicilin, kanamycin, PMSF, IPTG, Tris, MgCl${}_{2}$, HCl, NaOH, EDTA, glacial acid were products of Carl Roth (Karlsruhe, Germany). Coomassie Brilliant Blue G-250 was from Kodak (Rochester, NY, USA). DEAE-celulose (DE-52), Whatman 3MM, were products of Whatman (Maidstone, United Kingdom). Phenyl-Sepharose CL-4B was a product of GE Healthcare (Chicago, IL, USA). SnakeSkin™ Dialysis Tubing, 10K MWCO dialysis bags, were from Thermo Fisher Scientific (Waltham, MA, USA). [5-${}^{3}$H]dUMP was a product of Moravek Biochemicals (Brea, California, USA). Activated carbon Norit SX-2 and TCA were from POCH (Gliwice, Poland). N${}^{4}$-OH-dCMP (N4) was synthesised as previously described [43]. All solutions of substrate, inhibitors, cofactors, the WT mTS enzyme and its mutants were prepared in 50 mM PBS (phosphate buffer saline) with 10 mM β-mercaptoethanol (EtSH) added prior to experiments.

### 4.2. Expression and Purification of Thymidylate Synthase and Its Mutants

Site-directed mutagenesis was performed using GeneArt Site-Directed Mutagenesis System (Thermofisher Scientific, Waltham, MA, USA). Recombinant thymidylate synthase protein and its mutants (H190A and W103G) were over-expressed and purified as previously described [72,73,74], with some modifications. Phosphatase inhibitors (50 mM NaF, 5 mM Na-pyrophosphate, 0.2 mM EGTA, 0.2 mM EDTA and 2 mM Na${}_{3}$VO${}_{4}$) were present in the purification buffers. Purification process was held on ice or in a cooler (4 ${}^{\circ }$C). Purified TS preparation was separated into phosphorylated (P${}_{i}$+) and non-phosphorylated (P${}_{i}$-) fractions using a metal oxide/hydroxide affinity chromatography (MOAC) on Al(OH)${}_{3}$ beads [75]. The concentrations of purified WT mTS (non-phosphorylated) and its mutants were established by absorption measurements at $\lambda_{obs}$ = 280 nm. Activity of enzymes was determined with isotopic or spectroscopic assays described in Refs. [76,77,78].

### 4.3. Buffers and Solutions

For herein described experiments, Na${}^{+}$/K${}^{+}$ phosphate buffer (phosphate-buffer saline, PBS) at a concentration of 50 mM was prepared in twice distilled water. The 2M PBS stock solution was prepared from NaCl, KCl, Na${}_{2}$HPO${}_{4}$ and KH${}_{2}$PO${}_{4}$, and its pH was adjusted to 7.4. For the experimental investigations, the buffer was diluted 40× to a final concentration of 50 mM and β-mercaptoetanol (EtSH) was added prior to measurements to a 10 mM final concentration. Concentrations of the WT mTS enzyme and its mutants were kept in the range of 2–3.5 μM and of the nucleotides in the range of 4–7 μM (ratio 1:2 in favour of the nucleotide).

### 4.4. Fluorescence Spectroscopy

*Steady-state* fluorescence emission and excitation spectra were measured with a Spex (Edison, NJ, USA) FluoroMax spectrofluorimeter equipped with a xenon lamp, working in the photon counting mode, with 4 nm spectral resolution for excitation and emission and the signal-to-noise correction mode switched on. Fluorescence spectra were corrected for the background emission of the buffers, sample’s background and the inner filter effect (Equation (2)):

(2) $$G={\mathrm{antilog}}_{10}\left(\frac{\Delta A_{ex}+\Delta A_{em}}{2}\right)$$

where $\Delta A_{ex}$ and $\Delta A_{em}$ are absorption changes due to addition of molecules, at the excitation and emission wavelengths, respectively.

Corrected fluorescence spectra were normalised to unity by dividing each value by the maximum value of the emission:

(3) $$I_{norm}\left(\lambda \right)=I\left(\lambda \right)/I_{max}$$

where $I_{norm}\left(\lambda \right)$ is the normalised intensity at each wavelength, I(λ) is the measured value of the emission intensity at each wavelength and $I_{max}$ is the emission intensity at the maximum.

### 4.5. Multi-Dimensional Fluorescence Spectroscopy

Multi-Dimensional Fluorescence (MDF) spectroscopy can be implemented via two measurements: emission-excitation matrices (EEM) or Total Synchronous Fluorescence Spectroscopy (TSFS) both of which can provide a complete picture of the steady-state emission [79]. When EEM or TSFS data is analysed using PARAFAC (Parallel Factor Analysis) one can sometimes extract the contributions of individual fluorophores from mixtures. This can facilitate the resolution of overlapped emission spectra [80].

#### 4.5.1. Instrumentation

Polarized Total Synchronous Fluorescence Spectra (pTSFS) were collected at 20 ${}^{\circ }$C using a Cary Eclipse Spectrophotometer (Agilent Technologies, Santa Clara, CA, USA), fitted with bespoke dual wire-grid polarisers to enable steady-state anisotropy measurements in the UV region [81] and a temperature-regulated multi-cell holder. Emission was collected in TSFS mode. pTSFS data were collected over an excitation range of $\lambda_{exc}$ = 260–320 nm at varying wavelength offsets of 20–160 nm (i.e., corresponding to an emission range $\lambda_{em}$ = 300–480 nm) with 2 nm increments for both axes. Samples were excited along the short axis (4 mm), and emission was collected along the long axis (10 mm) of the cuvette. Excitation and emission monochromators’ split widths were 10 nm, the scan rate was 9600 nm/min, and the photomultiplier tube (PMT) detector voltage was set to 700 V.

#### 4.5.2. Data Pre-Processing and Analysis

All data analysis was conducted using MatLab (ver.9.1.0, MathWorks, Natick, MA, USA), PLS toolbox 8.2.1 (Eigenvector Research Inc., Manson, WA, USA), and in-house written codes. TSFS measurements were used in preference to EEM in order to minimise the Rayleigh scattering contamination when using wavelength offsets of Δλ≥ 20 nm [80]. The spectra were recorded with polarisers in vertical–vertical orientation (parallel polarised) as TSFS spectra. The TSFS spectra were subjected to Raman scattering minimisation by blank subtraction. Inner filter effect correction was not necessary due to a negligible optical density of the samples (Abs = 0.04 to 0.1, at 280 nm, 4 mm pathlength). Next, TSFS datasets were transformed from a non-trilinear TSFS layout to a trilinear EEM (emission-excitation matrices) layout for chemometric data analysis [82,83] and are hereafter designated t-EEM [84]. Interpolation was applied in order to handle the area with no experimentally acquired spectral information in the t-EEM layout [80]. Second-order scatter was also corrected via interpolation [85]. t-EEM data were smoothed using Savitzky-Golay smoothing in order to reduce a noise. The pre-processed data from 20 samples were arranged into a three-dimensional array (20 × 101 × 31) before the chemometric analysis.

### 4.6. Fluorescence Lifetime Spectroscopy

The use of a multi-anode detector with a TCSPC (time-correlated single photon counting)-based lifetime spectrometer and pulsed UV excitation enables the study of wavelength-dependent changes in Trp lifetimes.

#### 4.6.1. Instrumentation

The fluorescence lifetimes were recorded with a spectrometer assembled to measure intrinsic protein fluorescence in the 260 to 400 nm range (for a precise description, see [20]). Briefly, the excitation pulses were generated by a high-power super-continuum laser (SMHP-60.4 from Leukos, Limoges, France), coupled to a frequency doubling unit (BOX-UVgen2, Leukos, Limoges, France). The repetition rate of the laser was set to 30 MHz, and the excitation wavelength was fixed at 280 nm. The light coming out of the frequency doubling crystal, linearly polarised as it passed through a Glan-Thompson polariser, was focused in a temperature-controlled cuvette holder from Quantum Northwest (TLC-50F, Liberty Lake, WA, USA). In this experiment, samples were measured at 15 ${}^{\circ }$C in 100 μL UV fused quartz cuvettes (ThorLabs, Newton, NJ, USA). The cuvettes had 0.4 × 1 cm dimensions, with excitation along the short axis. The emitted light was collected through a broad-band anti-reflecting collimating lens (Balboa Scientific, Costa Mesa, CA, USA) and coupled to the detector through a multimode FC fibre in order to depolarise the emission. The detector, a 16 channel multi-anode photomultiplier with a bi-alkali photocathode from Becker & Hickl (Berlin, Germany, model PML-16-C-0), was optimised for 300–600 nm region, and controlled by a DCC100 card also from Becker & Hickl (Becker & Hickl).

In total, 16 decay curves were collected simultaneously, starting from channel 1 at 300 nm, and finishing at channel 16 at 500 nm, each channel being 12.5 nm apart. The laser power at the sample was ∼1.8–1.9 μW, which enabled us to record signals above 20k counts in the maximum (in the channel of the highest intensity) for samples in μM concentration range in times of 90 to 240 min, depending on their emission intensity. All the lifetime data presented were fitted to a $\chi^{2}$ value of between 1 and 1.6 for the channels of maximum intensity.

#### 4.6.2. Data Analysis

Analysis of decay parameters was performed with SPCImage software, provided by Becker & Hickl [86]. The instrument response function (IRF) was calculated from recorded data of the fluorescence decay curves for each emission channel. To calculate the intensity weighted lifetime, decay data were fitted with a sum of one, two or three exponential decays according to the equations in ref. [86].

### 4.7. Electrostatic Potential Computations

The methodology described below was used to determine the electrostatic potential (E.P.) of tryptophan residues in their local environment in mouse thymidylate synthase and its binary complexes. It is known that Trp emission is sensitive to the environment polarity and might change drastically, i.e., from short-wavelength emission in hydrophobic to long-wavelength emission in highly polar environments. Hence, to correlate experimental results and hypothesis based on them (Section 2.1 and Section 2.2) we have used the Advanced Poisson–Boltzmann Solver (APBS) method to calculate E.P. for all Trp residues.

Electrostatic potential computations for the crystal structures of mouse thymidylate synthase alone (mTS, PDB ID: 3IHI) [44] and in binary complexes with dUMP (PDB ID: 4E5O) [44] and N${}^{4}$OH-dCMP (PDB ID: 4EIN) [16] have been performed with continuum electrostatic calculations using Adaptive Poisson–Boltzmann Solver (APBS) v. 1.5 [87,88]. First, the crystal structures were prepared for APBS computations with PDB2PQR v. 2.1.1 [89], enabling the estimation of titration states and adequate protonation of molecules and the generation of pqr files with atomic charges and radii. APBS computations were carried out with the linearised PB equation with a solvent probe radius of 1.4 Å, surface sphere density of 10 gridpoints/Å${}^{2}$ and surface tension of 0.105 N/m. Temperature was set to 298.15 K, ionic strength to 0.15 M in monovalent salt and dielectric constants for solute (protein and ligands) and solvent to 2.0 and 78.54, respectively. A single Debye–Hückel boundary condition was applied. Atomic charges and radii for the protein were taken from the ff14sb amber force field [90], while atomic charges for ligands have been calculated with the am1-bcc method [91,92].

### 4.8. Tryptophan Accessibility

For each tryptophan residue in mTS (PDB ID: 3IHI) and its binary complexes with dUMP and N${}^{4}$-OH-dCMP (PDB ID: 4E5O and 4EIN, respectively), a solvent exposure was calculated with the GETAREA online software (http://curie.utmb.edu/getarea.html, accessed on January 2020) based on the Fraczkiewicz work [30]. This procedure was repeated for nine enzymes (44 Trp residues) studied in [36]. Obtained values were compared with the Acc parameter (accessibility, solvent exposure) from [36]. It appeared that in 90% of cases, values calculated with GETAREA were higher than Acc by approx. 50%. Hence, a rough estimation of Acc for tryptophan residues in 3IHI, 4E5O and 4EIN, by dividing values calculated with GETAREA by a factor of 1.5 was made.

### 4.9. mTHF-Dependent Covalent N${}^{4}$-OH-dCMP Binding by the Enzyme

The filter paper disc method was used to test formation of a denaturation—resistant, thus covalently bound nucleotide—TS complex, resulting from the reaction between the enzyme, N${}^{4}$-OH-[2-${}^{14}$C]dCMP and meTHF [15]. Following incubation, a part of the reaction mixture was deposited on a filter paper disc that was immediately immersed in 10% TCA. Washing with TCA solution, ethyl ether–ethanol mixture and ethyl–ether followed. Finally, the disc was dried, placed in the scintillation vial and counted.

## 5. Conclusions

In the present work, we studied properties and interactions of mouse thymidylate synthase (mTS) and its two mutants with with substrate (dUMP) and selected inhibitors using several emission-based spectroscopic methods as well as theoretical calculations. These methods, in particular multi-channel time-resolved spectroscopy, advanced techniques of spectral decomposition of fluorescence data and the Advanced Poisson–Boltzmann Solver method, proved to be powerful tools for detection of differences in interactions of the enzyme with ligand and enzyme property determination.

The experimental results demonstrated unique spectral features of Trp103 in mTS and its key role in the binding of ligands. The behaviour of this Trp residue can be explained on the basis of theoretical calculations. The latter showed that the distribution of the electrostatic potential (E.P.) around this particular tryptophan is significantly different than the other tryptophans in the enzyme. At the same time, comparison of the experimental and theoretical results indicated the importance of the distribution of E.P. (and the effect of His 190 on the E.P. distribution), for the properties of the enzyme, e.g., activity.

Studies of complexes of mTS and its mutants with dUMP, FdUMP and N${}^{4}$-OH-dCMP revealed that the E.P. seems to be a much more important factor in enzyme interactions with dUMP and FdUMP than with N4. This difference between dUMP/FdUMP and N${}^{4}$-OH-dCMP might be crucial in the explanation of the mechanism of the N${}^{4}$-OH-dCMP-driven “abortive reaction” of mTS. Of interest is that a lack of His190 (in the H190A mutant) seems to enhance the *syn-imino* N${}^{4}$-OH-dCMP binding, as suggested by the TCSPC results reflecting a different alignment of the N4-OH group. In accord, based on structural analyses and the H190A mutant capacity to form a denaturation-resistant complex with N${}^{4}$-OH-dCMP in the meTHF-dependent reaction, His190 is apparently responsible for a strong preference of the enzyme active center for the anti-rotamer of the *imino* inhibitor form.

## Figures and Tables

**Table 1:** Parameters describing local environments of tryptophans in both subunits of mTS (PDB ID: 3IHI), mTS + dUMP ((PDB ID: 4E5O) and mTS + N${}^{4}$-OH-dCMP (PDB ID: 4EIN). Acc—accessibility of a residue to the solvent, Den2—number of neighbouring atoms in a distance < 7.5 Å from the indole ring, E.P.—electrostatic potential values over the benzene and pyrrole rings of indole, Δ—the change of E. P. from the benzene to the pyrrole ring.

Parameter	Trp75	Trp84	Trp103	Trp133	Trp176
mTS (3IHI)					
Acc	12.1 (II)	0.9 (S)	7.1 (I)	1.14 (S)	0.8 (S)
	12.1 (II)	0.86 (S)	5.9 (I)	1.2 (S)	0.65 (S)
Den2	110 (II)	133 (I)	106 (II)	143 (S)	126 (I)
	111 (II)	137 (I)	121 (I/II)	145 (S)	125 (I)
E.P.	2.4 → 7.2	2.1 → 1.5	10 → 5.3	16.85 → 17.65	2 → 3
	2.7 → 7.2	0.1 → 1	9 → 4	21 → 22	2 → 4.5
Δ	+4.8	−0.6	−4.7	+0.8	+1
	+4.5	+0.9	−5	+1	+2.5
mTS + dUMP (4E5O)					
Acc	11.7 (II)	1.3 (S)	7.15 (I)	1.2 (S)	1.6 (S)
	7.9 (I/II)	0.9 (S)	7 (I)	1.3 (S)	1.1 (S)
Den2	119 (II)	134 (I)	128 (I)	142 (S)	132 (I)
	111 (II)	137 (I)	127 (I)	140 (S)	131 (I)
E.P.	0.8 → 6.4	−3.6 → 2	3.6 →−1.6	6.9 → 7.1	−0.6→ 1.6
	1.3 → 6.4	1.4 → 2.65	2.45 →−0.6	19.45 → 20	0.75 → 1.65
Δ	+5.6	+5.6	−5.2	+0.2	+2.2
	+5.1	+1.25	−3.05	+0.55	+0.9
mTS + N4 (4EIN)					
Acc	12.1 (II)	0.64 (S)	7.6 (I/II)	2.8 (I)	0.7 (S)
	12 (II)	0.86 (S)	8.7 (I/II)	2.7 (I)	0.8 (S)
Den2	109 (II)	138 (I)	121 (I/II)	142 (S)	127 (I)
	113 (II)	134 (I)	118 (II)	140 (S)	126 (I)
E.P.	1.4 → 5.6	−1.7 →−0.2	2.2 →−3	19.5 → 20.3	2.4 → 5.2
	1.4 → 5.6	−0.6 → 0	2 →−1.4	24.6 → 25.4	−1.7 → 0.05
Δ	+4.2	+1.5	−5.2	+0.8	+2.8
	+4.2	+0.6	−3.4	+0.8	+1.75

**Table 2:** Average fluorescence decay time ($\tau_{av}$), decay times of its components ($\tau_{n}$) in [ns] and fractions of components (a${}_{n}$) in [%] determined for mTS, H190A, and W103G for different emission wavelengths. The $\chi^{2}$ values calculated are in the range of 1–1.6.

Channel	mTS			H190A			W103G		
ine	$\tau_{av}$	$\tau_{n}$	a${}_{n}$	$\tau_{av}$	$\tau_{n}$	a${}_{n}$	$\tau_{av}$	$\tau_{n}$	a${}_{n}$
no. 1	2.23 ± 0.04	1.3 ± 0.1	80 ± 3	2.3 ± 0.1	1.33 ± 0.03	83 ± 1	2.36 ± 0.02	1.58 ± 0.02	75.6 ± 0.5
290–303		6.2 ± 0.7	20 ± 3		7.3 ± 0.6	17 ± 1		5.1 ± 0.2	22.4 ± 0.5
no. 2	1.93 ± 0.04	1.32 ± 0.03	86 ± 1	1.89 ± 0.01	1.32 ± 0.01	85.2 ± 0.3	2.55 ± 0.01	1.42 ± 0.03	55.7 ± 0.4
303–316		5.5 ± 0.3	14.5 ± 0.9		5.2 ± 0.1	14.8 ± 0.3		3.97 ± 0.02	44.3 ± 0.4
no. 3	2.8 ± 0.1	1.46 ± 0.04	69.5 ± 0.3	2.78 ± 0.03	1.47 ± 0.03	70.7 ± 0.1	3.14 ± 0.01	2.0 ± 0.2	50 ± 7
316–329		5.9 ± 0.1	30.5 ± 0.3		5.95 ± 0.02	29.3 ± 0.1		4.3 ± 0.1	50 ± 7
no. 4	4 ± 0.1	1.63 ± 0.03	50.6 ± 0.3	3.7 ± 0.02	1.65 ± 0.01	46.9 ± 0.2	3.50 ± 0.01	2.46 ± 0.01	50.3 ± 0.5
329–343		6.3 ± 0.1	49.4 ± 0.3		6.03 ± 0.04	53.1 ± 0.2		4.56 ± 0.01	49.7 ± 0.5
no. 5	5.0 ± 0.1	1.8 ± 0.1	31 ± 1	4.9 ± 0.1	1.6 ± 0.1	30 ± 1	3.90 ± 0.01	2.93 ± 0.01	59 ± 2
343–356		6.0 ± 0.2	19 ± 1		6.0 ± 0.2	30 ± 18		4.87 ± 0.05	41 ± 2
		6.6 ± 0.2	50 ± 1		6.5 ± 0.2	40 ± 17			
no. 6	6.0 ± 0.2	5.0 ± 0.1	60 ± 2	6.09 ± 0.01	5.0 ± 0.2	58 ± 2	3.90 ± 0.01	3.3 ± 0.5	70 ± 1
356–369		7.6 ± 0.2	40 ± 2		7.6 ± 0.1	42 ± 2		5.26 ± 0.02	30 ± 1
no. 7	6.5 ± 0.1	-	100	6.53 ± 0.01	-	100	4.03 ± 0.02	3.48 ± 0.01	74 ± 2
369–382								5.6 ± 0.2	26 ± 2
no. 8	6.7 ± 0.1	-	100	6.8 ± 0.1	-	100	4.22 ± 0.02	-	100
382–395									
no. 9	6.7 ± 0.1	-	100	6.9 ± 0.1	-	100	4.30 ± 0.02	-	100
395–408									
no. 10	6.9 ± 0.1	-	100	6.91 ± 0.05	-	100	4.47 ± 0.03	-	100
408–421									
no. 11	6.8 ± 0.2	-	100	6.9 ± 0.1	-	100	4.71 ± 0.02	-	100
421–434									

## Data Availability

Not applicable.

## Abbreviations

The following abbreviations are used in this manuscript:

ARMES	Anisotropy-Resolved Multi-Dimensional Emission Spectroscopy
Asn/N	asparagine
C	-cysteine
CT	charge transfer
DHF	dihydrofolate
dTMP	deoxythymidine monophosphate
dUMP	deoxyuridine monophospate
*Ec*TS	*E. coli* thymidylate synthase
EEM	emission-excitation matrices
E.P.	electrostatic potential
FdUMP	5- fluoro deoxyuridine monophospate
HH	horizontal–horizontal
His/H	histidine
hTS	human thymidylate synthase
HV	vertical–horizontal
KIE	kinetic isotop effect
K${}_{m}$	Michaelis–Menten constant
${}^{1}$L${}_{a}$	emitting excited state of tryptophan
mTHF	R-N${}^{5,10}$ methylenetetrahydrofolate
mTS	mouse thymidylate synthase
N4	N${}^{4}$-OH-dCMP
PARAFAC	Parallel Factor Analysis—a multi-way method originating from psychometrics
PMT	photomultiplier tube
pTSFS	Polarised Total Synchronous Fluorescence Spectra
QM	quantum mechanics
QY	quantum yield
TCSPC	Time-Correlated Single Photon Counting
Trp/W	tryptophan
TS	thymidylate synthase
Tyr	tyrosine
VH	vertical–horizontal
VV	vertical–vertical

## Appendix A

**Table A1:** Summary of the values of total variance explained by the model (variance explained (%)), % of variance captured by PARAFAC analysis for each component (PaC1/PaC2${}_{\lambda_{ex}/\lambda em}$), CONCORDIA value (CONCORDIA (%)) and split-half analysis [28]. Presented values are calculated for the two-component model of MDF analysis.

PaC1${}_{\lambda_{\mathrm{ex}}/\lambda \mathrm{em}}$ (nm)	PAC1 Fit Model (%)	PaC2${}_{\lambda_{\mathrm{ex}}/\lambda \mathrm{em}}$ (nm)	PaC2 Fit Model (%)	Variance Explained (%)	CONCORDIA (%)	Split-Half Analysis
280/326	30.2	282/356	69.8	99.7	98.4	78.6

**Table A2:** Summary of the values of total variance explained by the model (variance explained (%)), % of variance captured by PARAFAC analysis for each component (PaC1/PaC2/PaC3${}_{\lambda_{ex}/\lambda em}$), CONCORDIA value (CONCORDIA (%)) [28] and split-half analysis. Presented values are calculated for the three-component model of MDF analysis.

PaC1${}_{\lambda_{\mathrm{ex}}/\lambda \mathrm{em}}$ (nm)	PAC1 Fit Model (%)	PaC2${}_{\lambda_{\mathrm{ex}}/\lambda \mathrm{em}}$ (nm)	PaC2 Fit Model (%)	PaC3${}_{\lambda_{\mathrm{ex}}/\lambda \mathrm{em}}$ (nm)	PaC3 Fit Model (%)	Variance Explained (%)	CONCORDIA (%)	Split-Half Analysis
284/362	68.9	278/334	25.68	294/334	5.4	99.9	−**702.25**	11.1
